# Characterization of ROMK cellular heterogeneity along the mouse kidney thick ascending limb

**DOI:** 10.1007/s00424-025-03086-4

**Published:** 2025-05-13

**Authors:** Christian Keller, Rui Ramos Santos, Wouter H. van Megen, Johannes Loffing

**Affiliations:** 1 Institute of Anatomy, University of Zurich, Winterthurerstrasse 190, CH- 8057 Zurich, Switzerland; 2 PhD Program Biomedicine, University of Zurich, Zurich, Switzerland; 3 Ophthalmology Clinic, City Hospital Zurich, Zurich, Switzerland; 4 Spross Research Institute, Zurich, Switzerland

**Keywords:** Kidney, Thick ascending limb, ROMK, Claudin, Multiplexed imaging

## Abstract

The renal thick ascending limb (TAL) plays a key role in water and ion homeostasis. Apical potassium secretion via the renal outer medullary potassium channel (ROMK) is essential for transepithelial sodium reabsorption via the furosemide-sensitive Na-K-2Cl-cotransporter and creates the electrochemical gradient for paracellular ion transport through Claudin tight junction proteins. Interestingly, the TAL exhibits transcriptomic heterogeneity and variable apical ROMK abundance. Single-cell RNA sequencing suggests that the cortical TAL consists of at least three distinct cell types, but whether ROMK distribution aligns with these types remains unclear. We analyzed perfusion-fixed mouse kidneys using RNAscope in situ hybridization (ISH), iterative indirect immunofluorescence imaging (4i multiplexing), and machine learning. ROMK mRNA expression was seen in all TAL cells. In contrast, apical ROMK protein abundance was found on almost all macula densa (MD) cells but was heterogeneous along the rest of the TAL. In the remaining TAL, only about 60% of the TAL cells had strong apical ROMK staining, while 40% lacked apical ROMK but showed weak perinuclear signals. ISH revealed that apical ROMK-positive cells express Ptger3 mRNA, whereas apical ROMK-negative cells express Foxq1 mRNA. Multiplexing analysis showed that ROMK-positive cells form Claudin-10b-positive tight junctions, while ROMK-negative cells form Claudin-16/19-positive junctions and express basolateral Kir4.1. Despite universal ROMK mRNA expression, apical ROMK distribution aligns with molecularly distinct TAL cell types. This unique ROMK expression pattern suggests functional heterogeneity for ROMK along the TAL.

## Introduction

The renal thick ascending limb (TAL) plays a crucial role in blood pressure regulation, urine concentration, and maintaining systemic electrolyte balance through regulated ion reabsorption [4, 17]. The ability of the TAL to reabsorb ions is driven by a coordinated transport system, including the apical sodium-potassium-chloride cotransporter 2 (NKCC2) and the renal outer medullary potassium channel (ROMK). NKCC2 facilitates transcellular reabsorption of sodium, potassium, and chloride from the tubular lumen. Its activity is supported by ROMK which recycles potassium back into the lumen. Together with basolateral chloride extrusion through the ClC-Kb chloride channel and its accessory protein Barttin, NKCC2 and ROMK are responsible for establishing a lumen-positive electrical gradient [30]. This subsequently drives paracellular calcium and magnesium reabsorption through pore-forming claudins, including Claudin-16 (Cldn16) and Claudin-19 (Cldn19) [1, 30]. The critical importance of electrolyte transport in the TAL is underscored by genetic disorders such as Bartter syndrome, where mutations in genes encoding NKCC2 (*SLC12A1*), ROMK (*KCNJ1*), ClC-Kb (*CLCNKB*), or Barttin (*BSND*) result in severe renal water loss, electrolyte imbalance, and hypokalemic metabolic alkalosis [29]. Additionally, mutations in *CLDN16* and *CLDN19* cause familial hypomagnesemia with hypercalciuria and nephrocalcinosis [38].

Traditionally, the TAL has been considered a relatively uniform structure in terms of its cellular and functional properties. However, the presence of the macula densa (MD) as a specialized and histologically distinct cell population within the TAL already suggests some degree of cellular heterogeneity. MD cells are taller and have more densely packed nuclei than the surrounding TAL cells [5]. The ability of the MD to sense intratubular sodium chloride concentrations is critical for maintaining a constant single-nephron glomerular filtration rate upon changes in blood pressure through initiating tubuloglomerular feedback (TGF) [18]. Additionally, the salt-sensing capacity of the MD is important for systemic blood pressure regulation by controlling the release of renin from the glomerular afferent arterioles, which serves as the first step of the renin-angiotensin-aldosterone system (RAAS) [32].

Remarkably, morphological and molecular biology techniques have revealed significant additional cellular heterogeneity along the TAL [2, 9]. Histological studies in rats and hamsters identified two morphologically distinct TAL cell types that are intermingled with each other but characterized by either short or long microvilli [2, 37]. Furthermore, immunohistochemical studies demonstrated a heterogeneous composition of tight junctions (TJ) in the TAL, consisting of either Claudin-10b (Cldn10b) or Cldn16 and Cldn19 [27]. Recent single-cell RNA sequencing (scRNAseq) studies have provided additional evidence for the existence of at least three different cell populations in the cortical TAL which includes the MD cells and two cell types along the rest of the cortical TAL [9]. The first cell population (named C5) is characterized by the expression of marker mRNAs such as *Cldn16*, *Foxq1*, and *Kcnj10*, while the second cell population (named C6) expresses markers such as *Cldn10b* and *Ptger3* but notably lacks *Kcnj10*. The *Foxq1* gene encodes the Forkhead Box Q1 transcription factor, which is involved in epithelial-to-mesenchymal transition, cell proliferation, tumor development [20, 22], and was suggested to improve renal outcomes following sepsis-induced acute kidney injury [45]. The Kcnj10 gene encodes the inwardly rectifying potassium channel Kir4.1, which forms a functional heteromer with Kir5.1 (encoded by KCNJ16) and plays a critical role in potassium transport in renal epithelial cells and the brain. In humans, mutations in KCNJ10 (Kir4.1) are responsible for EAST syndrome (also known as SeSAME syndrome), characterized by epilepsy, ataxia, sensorineural deafness, and salt-wasting tubulopathy [7, 35, 41]. The Ptger3 gene encodes the EP3 receptor, one of four prostaglandin E2 (PGE2) receptors expressed in various tissues. In the kidney, EP3 is believed to contribute to the regulation of urinary concentration [8]. However, a recent study using kidney tubule-specific EP3 knockout mice found no significant impact on water homeostasis, even under various experimental challenges [16]. In addition to the two TAL cell types, the scRNAseq studies did also confirm that MD cells form a distinct TAL cell population (named C14) characterized by, for example, a strong expression of the nitric oxidase 1 (NOS1) [9]. A similar cellular heterogeneity of the TAL was observed by others using single-nucleus RNA sequencing (snRNA-seq) on mouse and human kidneys [19, 42].

Interestingly, heterogeneity in the apical expression pattern of ROMK in the TAL has also been observed in rats, with some cells showing strong apical staining while others displaying no visible ROMK immunostaining [26, 43, 46]. However, it is currently unclear if the difference in apical ROMK staining also reflects different cell types. In this study, we therefore used multiplex immunostaining combined with supervised machine learning to further define the cellular heterogeneity of the TAL, with a special emphasis on the immunohistochemical abundance of ROMK, to determine whether the previously described heterogeneity in ROMK expression correlates with one or more of the identified TAL cell types.

## Materials and methods

### Mice

Most experiments were performed on kidneys from adult (2-4 months old) male C57Bl/6 J and 129S/SvEv mice. Furthermore, we analyzed archived kidneys from male and female wildtype and gene-modified mice (B6;129S and NMRI) as indicated in Table 1. Mice were kept on standard lab chow containing 0.21% Na^+^ and 0.97% K^+^ (Cat.: E15430 - 047, Ssniff, Soest, Germany). Only mice with a constitutive deletion of the NaCl cotransporter (NCC^−/−^) and their wildtype littermates (NCC^+/+^) were kept on standard lab chow containing 0.2% Na^+^ and 0.78% K^+^ (Cat.: 3433, KLIBA NAFAG, Kaisersaugst, Switzerland). Animal experiments were performed according to Swiss law and were approved by the local veterinary authority (license numbers: ZH192/2007, ZH185/2017, ZH80/2022).

**Table 1:** Comparative quantification of apical ROMK along the TAL

Age	Sex	Strain	Genotype	*n*	ROMK area/NKCC2 area [%]			
					Cortex	MD	OMOS	OMIS
4 months	m	C57Bl/6 J	NCC^+/+^ - control	5	61.64 ± 2.04	91.11 ± 2.53	57.81 ± 1.55	65.13 ± 3.06
4 months	m	C57Bl/6 J	NCC^−/−^	5	65.45 ± 0.99	94.76 ± 0.73	56.51 ± 1.11	62.70 ± 3.37
6-7 months	m	B6;129S	CnB1^fl/fl^	4	63.33 ± 1.54	92.66 ± 1.63	61.12 ± 2.76	68.02 ± 2.55
6-7 months	m	B6;129S	NCC^cre^ × CnB1^fl/fl^	4	66.63 ± 6.61	91.13 ± 2.76	66.39 ± 5.70	64.44 ± 3.66
2-3 months	m	NMRI	Wild type	3	59.51 ± 1.89	95.79 ± 1.87	59.94 ± 1.36	60.64 ± 0.26
2-3 months	f	NMRI	Wild type	4	60.28 ± 2.30	93.57 ± 3.42	60.54 ± 1.66	61.98 ± 2.03

Data shown as mean ± standard error of mean. n, number of animals. m, male. f, female

### RNAscope combined with immunofluorescence

**Fig. 1:**
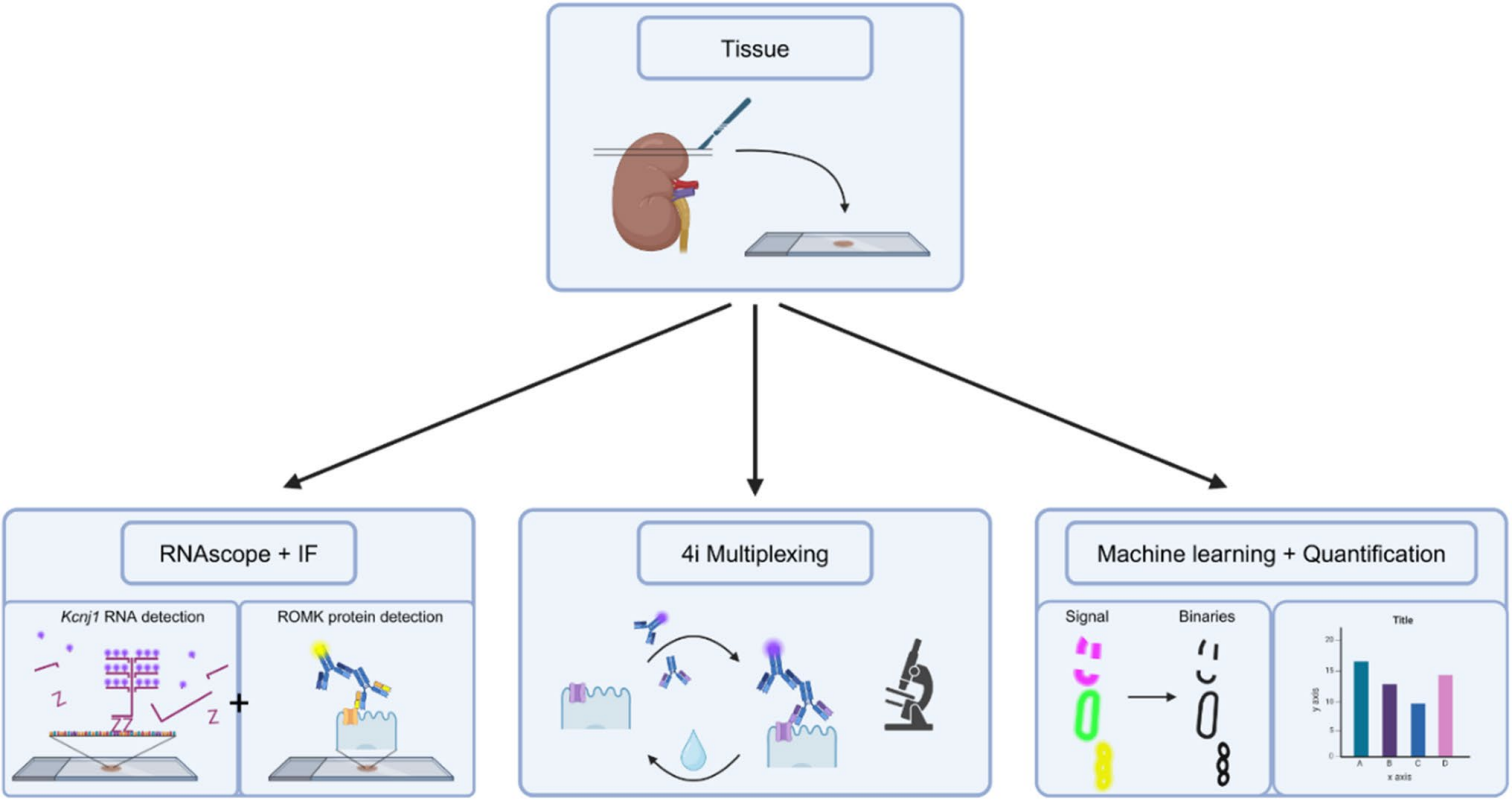
Imaging pipeline. Cryosections of mouse kidneys were placed on glass slides (top panel) and then processed for RNAscope detection of various mRNAs, combined with immunofluorescent detection of ROMK (lower left panel), for 4i multiplexing immunodetection of several antigens, including ROMK (lower middle panel), or for automated image analysis (lower right panel). For the latter, immunofluorescent stainings for ROMK, NKCC2, and NOS1 were converted to binaries using supervised machine learning (Ilastik) and then analyzed for automated quantification of the cell surface abundance of ROMK

The kidneys were fixed for 5 min with 3% paraformaldehyde (PFA) (Cat.: A3813, PanReac AppliChem, Chicago, US) in phosphate buffer (PB) (19 mM NaH_2_PO_4_ (Cat.: 71507, Sigma-Aldrich, St. Louis, US), 81 mM Na_2_HPO_4_ (Cat.: 71645, Sigma-Aldrich, St. Louis, US), 0.33 mM CaCl_2_ (Cat.: 12074, Sigma-Aldrich, St. Louis, US), pH = 7.3, mOsm = 290, Sucrose (Cat.: 84100, Merck Millipore, Burlington, US)) by retrograde perfusion via the abdominal aorta and subsequently rinsed with PB for another 5 min. After organ harvesting, the kidneys were cut into ~ 1-mm-thick slices, frozen in liquid propane, and stored at – 80 °C until further use. 9-μm-thick cryosections were placed on coated glass slides (Cat: 10149870, Epredia, Kalamazoo, US), temporarily stored in ice cold 3% PFA in phosphate-buffered saline (PBS) (~ 2.7 mM KCl (Cat.: 60130, Sigma-Aldrich, St. Louis, US), ~ 1.47 mM KH_2_PO_4_ (Cat.: 131509, PanReac AppliChem, Chicago, US), ~ 137 mM NaCl (Cat.: 71380, Honeywell Fluka, Charlotte, US), ~ 8.1 mM Na_2_HPO_4_ (Cat.: 71645, Sigma-Aldrich, St. Louis, US), pH = 7.3), and underwent epitope retrieval at 80 °C for 10 min in citrate buffer (10 mM citric acid (Cat.: 33114, Sigma-Aldrich, St. Louis, US, pH = 6.0)). The slices were washed twice with PBS and then dehydrated with a graded series of 50%, 70%, and 100% ethanol (Cat.: 20821.321, VWR, Radnor, US) for 5 min at RT. The slices were dried out, incubated with 0.3% hydrogen peroxide for 15 min at RT, and then washed twice with PBS. To preserve tissue antigenicity and contrary to recommendations by the supplier, no proteases were used for the RNAscope protocol. RNAscope probes (Table 2) were prewarmed to 40 °C for 10 min and then cooled down to RT before dilution in probe diluent (Cat.: 300041, ACD Bio, Newark, US). Subsequently, the slices were incubated with the diluted probes at 40 °C overnight. The next day, the slices were washed twice with washing buffer (WB) (Cat.: 310091, ACD Bio, Newark, US) and incubated with AMP1, AMP2, and AMP3 (Cat.: 323110, ACD Bio, Newark, US) for 30 min, 30 min, and 15 min, respectively, in the oven at 40 °C with two washes in between. The tissue was then incubated with HRP-CX (Cat.: 323110, ACD Bio, Newark, US) for 15 min at 40 °C in the oven, the slices were washed twice with WB, and the signal was developed by incubating with the dye (Table 2), diluted in TSA buffer (Cat.: 322809, ACD Bio, Newark, US), for 30 min at 40 °C in the oven. Afterwards, the slices were washed twice with WB, HRP was deactivated by HRP-blocker (Cat.: 323110, ACD Bio, Newark, US) for 15 min in the oven, slices were washed twice with WB, and once with PBS. Then, the tissue was postfixed with 2% PFA in PBS for 10 min at RT, washed twice with PBS, and quenched with 0.1 M glycine (Cat.: 071323, Biosolve, Dieuze, France) for 10 min at RT. The slices were then blocked with 5% v/v normal goat serum (NGS) in PBS/BSA/Triton 0.1% (PBS + 1% bovine albumin serum (BSA) (Cat.: A7906, Sigma-Aldrich, St. Louis, US) + 0.1%Triton-X (Cat.: X100, Sigma-Aldrich, St. Louis, US). The tissues were incubated with primary antibodies (Table 2) diluted in PBS/BSA/Triton 0.1% at 4 °C overnight. After washing thrice for 5 min with PBS/BSA/Triton 0.1%, the slices were then incubated with secondary antibodies (Table 2) and DAPI (1:500) (Cat.: D9542, Sigma-Aldrich, St. Louis, US) in PBS/BSA/Triton 0.1% for 2 h at RT. Following thrice washing for 5 min with PBS/BSA/Triton 0.1%, the slices were postfixed with 3% PFA in PBS and then mounted with Glycergel (Cat.: C0563, Dako, Glostrup, Denmark) with DAKBO (Cat.: D27802, Sigma-Aldrich, St. Louis, US) (Fig. 1).

**Table 2:** RNAscope probes and antibodies used for the different staining protocols

RNAscope						
RNAscope Probe	Dilution		Cat. / Ref.:	HRP	Dye	Dilution
C1-*Foxq1*	1/4		504504801, ACD Bio, Newark, US	HRP-C1	Cy5	1/500
C3-*Ptger3*	1/1'000		501831-C3, ACD Bio, Newark, US	HRP-C3	Cy5	1/1'000
C2-*Kcnj1*	1/1'000		818391-C2, ACD Bio, Newark, US	HRP-C2	Cy5	1/1'000
IF						
Prim. AB	Dilution		Cat. / Ref.:	Sec. AB	Dilution	Cat.
rb-ROMK	1/5'000		[31]	g-rb-AF555	1/500	A32732, Invitrogen, Waltham, US
g-ZO-1	1/500		33-9100, Invitrogen, Waltham, US	g-m-AF488	1/500	A11029, Invitrogen, Waltham, US
4i Multiplexing						
Cycle	Prim. AB	Dilution	Cat. / Ref.:	Sec. AB	Dilution	Cat.:
1	rb-ROMK	1/5'000	[31]	g-rb-AF488	1/500	111-545-144, Jackson ImmunoResearch, Baltimore, US
1	g-ZO-1	1/500	33-9100, Invitrogen, Waltham, US	g-m-AF555	1/500	A21424, Invitrogen, Waltham, US
2	rb-Cldn16	1/5'000	00216, BiCell Scientific Inc, St. Louis, US	dk-rb-AF647	1/500	711-605-152, Jackson ImmunoResearch, Baltimore, US
3	rb-Cldn10	1/5'000	00210, BiCell Scientific Inc, St. Louis, US	g-rb-AF555	1/500	A32732, Invitrogen, Waltham, US
4	rb-KCNJ10	1/5'000	APC035, Alomone Labs, Jerusalem, Israel	g-rb-AF488	1/500	111-545-144, Jackson ImmunoResearch, Baltimore, US
5	rb-Cldn19	1/5'000	00219, BiCell Scientific Inc, St. Louis, US	g-rb-AF647	1/500	111-605-144, Jackson ImmunoResearch, Baltimore, US
Simple IF						
Cycle	Prim. AB	Dilution	Cat. / Ref.:	Sec. AB	Dilution	Cat.:
1	rb-ROMK	1/5'000	[31]	dk-rb-AF647	1/500	711-605-152, Jackson ImmunoResearch, Baltimore, US
2	rb-NKCC2	1/5'000		g-rb-AF488	1/500	111-545-144, Jackson ImmunoResearch, Baltimore, US
2	m-NOS1-PE	1/50	sc-5302, Santa Cruz, Dallas, US			
Cycle	Prim. AB	Dilution	Cat. / Ref.:	Sec. AB	Dilution	Cat.:
1	rb-ROMK	1/5'000	[31]	dk-rb-AF647	1/500	711-605-152, Jackson ImmunoResearch, Baltimore, US
2	rb-NKCC2	1/5'000	[39]	g-rb-AF555	1/500	A32732, Invitrogen, Waltham, US
2	m-NOS1-AF488	1/50	sc-5302, Santa Cruz, Dallas, US			
Cycle	Prim. AB	Dilution	Cat. / Ref.:	Sec. AB	Dilution	Cat.:
1	rb-ROMK	1/5'000	[31]	dk-rb-AF647	1/500	711-605-152, Jackson ImmunoResearch, Baltimore, US
1	m-ZO-1	1/500	33-9100, Invitrogen, Waltham, US	g-m-AF488	1/500	A11029, Invitrogen, Waltham, US
2	rb-Cldn10	1/5'000	00210, BiCell Scientific Inc, St. Louis, US	g-rb-AF555	1/500	A32732, Invitrogen, Waltham, US
	Alt. Prim. AB	Dilution	Cat. / Ref.:	Sec. AB	Dilution	Cat.:
	rb-ROMK	1/500	APC-001, Alomone Labs, Jerusalem, Isreal	g-rb-Cy3	1/1'000	111-165-144, Jackson ImmunoResearch, Baltimore, US

### 4i multiplexing

For detection of several antigens in the same cryosection, we used a previously published protocol [10] with some modifications. Perfusion-fixed kidneys were cut into 4 pieces each and placed into 15% w/v sucrose for 2 h and subsequently into 30% w/v sucrose overnight. On the next day, the tissue pieces were embedded in O.C.T. compound (Cat.: 361603E, VWR Chemicals, Radnor, US), frozen with 2-methylbutane at – 40 °C and stored at – 80 °C until further use. Two 3-mm-hard plastic wells were glued into chambered cover glasses (Cat.: C1 - 1.5H-N, Cellvis, Mountain View, CA) with Twinsil 2-component silicon (Cat.: 1300 1000, Picodent, Wipperfürth, Germany). The resulting wells were coated with poly-d-lysine (Cat.: P6407 - 5MG, Sigma-Aldrich, St. Louis, US). 9-µm-thick cryosections were put into the chambered cover glasses and dried overnight to adhere to the cover glass. Antibody retrieval was performed at 90 °C for 20 min with citrate buffer (pH = 6.0). A cycle of staining, imaging, and elution of the tissue started with three 5-min washings with PBS, followed by blocking with 5% NGS and/or normal donkey serum (NDS) in PBS/BSA/Triton 0.2% (PBS + 1% BSA + 0.2% Triton-X). The tissue was incubated overnight with the primary antibodies diluted in PBS/BSA/Triton 0.1% (Table 2) at 4 °C, washed thrice for 5 min with PBS, and rinsed once with PBS/BSA/Triton 0.1%. The secondary antibodies (Table 2) and DAPI (1:500) diluted in PBS/BSA/Triton 0.1% were applied for 2 h at RT, after which the tissue was washed thrice for 5 min with PBS. Before adding freshly prepared imaging buffer (0.7 M N-Acetylcysteine (Cat.: A9165 - 25G, Sigma-Aldrich, St. Louis, US) in 0.2 M PB buffer, pH = 7.4), the tissue was rinsed again thrice with PBS. The samples were imaged with Leica DM6000 B (Table 2) at low exposure time and intensity to avoid photon-induced cross-linking. After imaging, the samples were washed thrice for 5 min with PBS, and then, the antibodies were eluted with three 10 min washes at RT with equal parts ddH_2_O and elution buffer (0.5 M Glycine, 3 M Urea (Cat.: U5378, Sigma-Aldrich, St. Louis, US), 3 M Guanidine hydrochloride (Cat.: 1302134 - 100MG, Sigma-Aldrich, St. Louis, US) in ddH_2_O, 0.07 M TCEP-HCl (Cat.: P1020 - 10G, Lucerna-Chem, Luzern, Switzerland), pH = 2.5). Between each staining cycle, tissue slices were studied microscopically to confirm that antibody complexes were eluted before the next staining was performed.

### Quantification of apical ROMK

Kidney sections were immunostained for ROMK, NKCC2, and NOS1 (Table 2) similar to the immunostaining protocol described for RNAscope combined with immunofluorescence. However, as ROMK and NKCC2 antibodies are both derived from rabbit, the slices were stained by consecutive immunolabeling cycles. The first cycle was done with a primary rabbit anti-ROMK antibody and a secondary AF647-conjugated anti-rabbit antibody, followed by a second cycle with a rabbit antibody against NKCC2 and an AF488-conjugated anti-rabbit secondary antibody, which resulted in a strong and specific ROMK signal from the AF647 fluorophore and for NKCC2 from the AF488 fluorophore. A very weak signal was also seen for ROMK coming from the AF488 fluorophore. However, this could be neglected for further imaging and analysis, as it got hidden in the strong NKCC2 signal. Tile scans of whole kidney slices were imaged with a Leica DM6000 B, and detected TALs were cropped and grouped by region (OMIS TAL, OMOS TAL, cortical TAL, MD cells). Using pixel classification from Ilastik, we converted the original images to probability maps and then to binaries. Using the binaries, we calculated the ratio of the apical area positive for ROMK vs. NKCC2 on a pixel-by-pixel basis (Fig. 1).

To assess the signal strength of apical ROMK, we used the abovementioned pipeline, additionally overlaid the generated binaries with the original images, and quantified the signal strength in those regions.

### Software

Microscope images were deconvoluted with standard and automatically calculated settings by the Huygens Software (Huygens Software, Scientific Volume Imaging), contrast adjusted, cropped by Fiji (Fiji, open source), and arranged by Affinity Designer 2 (Affinity Designer, Serif). Registration of the deconvoluted 4i multiplexed images was done by Fiji (Fiji, open source) and QuPath (QuPath, open source) using the DAPI signal as a reference. Graphics (Fig. 1 and Fig. 9) were designed by BioRender. Ilastik was used for pixel classification in the quantification of apical ROMK, and the generated data was statistically analyzed and arranged by GraphPad Prism 10 (GraphPad Prism, Prism Software).

### Microscopy

All immunofluorescent images were acquired using a Leica DM6000 B microscope (Leica, Wetzlar, Germany) equipped with a Scientific Complementary Metal–Oxide–Semiconductor (sCMOS) camera. Most images were acquired with the Leica DFC9000 GT camera (Leica, Wetzlar, Germany), except for the data on sex differences, which were obtained by the Leica K8 camera (Leica, Wetzlar, Germany). The images presented are representative of microscopic analyses from at least three animals, with the exception of 4i multiplexing, which was performed on kidneys from a single animal only.

### Statistics

Quantitative data (Table 1, Fig. 8) are presented as mean ± standard error of the mean, and statistical significance was assessed using a two-tailed unpaired t-test. A *P* value < 0.05 was considered to be statistically significant.

## Results

### Heterogeneous apical localization of ROMK in the mouse TAL

**Fig. 2:**
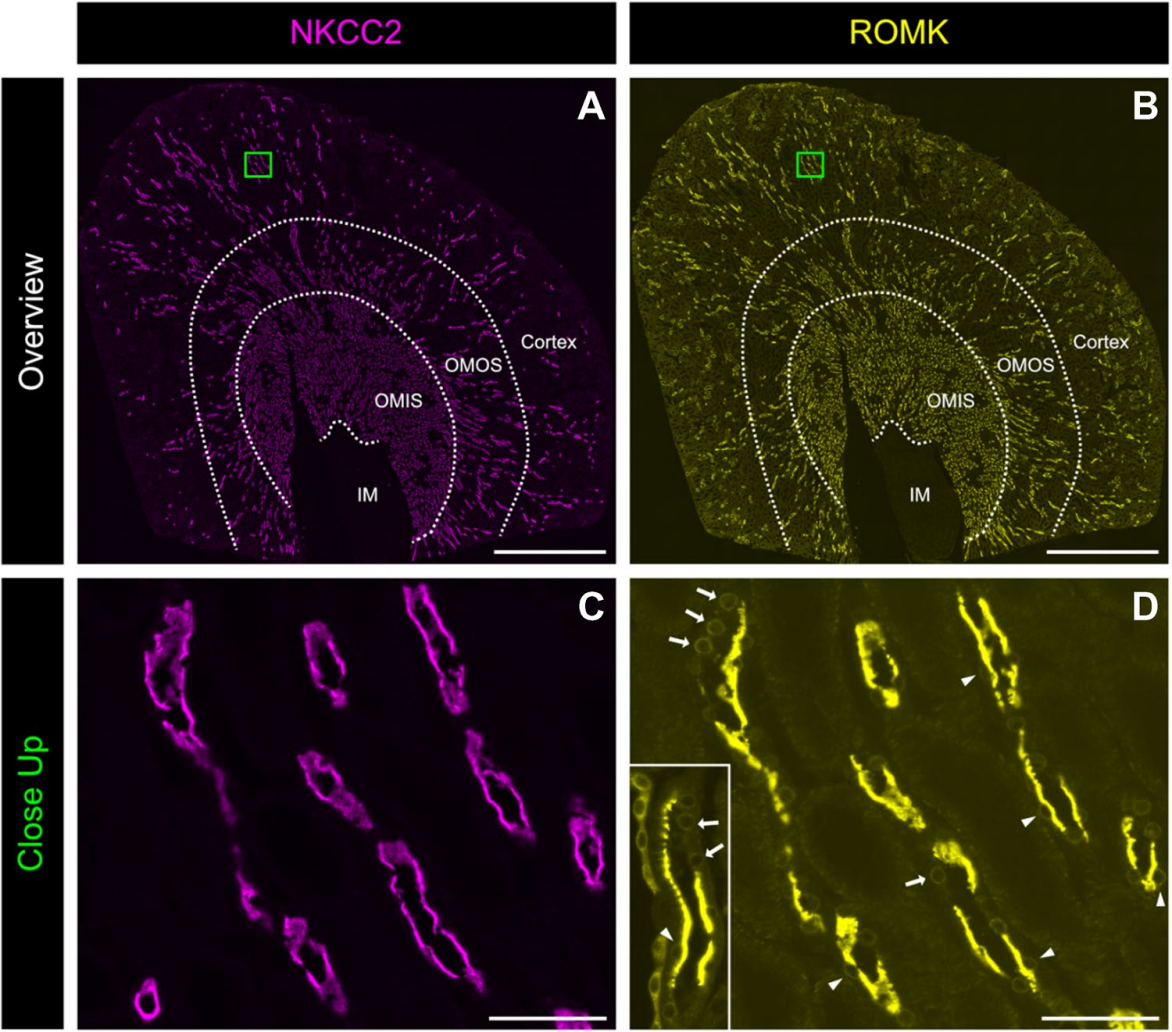
Co-immunodetection of NKCC2 and ROMK. **A**, **B** Overviews of immunohistochemical stainings with our previously published antibodies against NKCC2 and ROMK; kidney zones (cortex, OMOS, OMIS, and inner medulla (IM) are indicated by dotted lines. **C**, **D** Close-ups showing TALs stained with our previously published antibodies against NKCC2 and ROMK (Table 2); insert in **D** immunodetection of ROMK with the commercially available antibody from Alomone (Table 2); arrowheads – apical localization of ROMK; arrows – perinuclear localization of ROMK

**Fig. 3:**
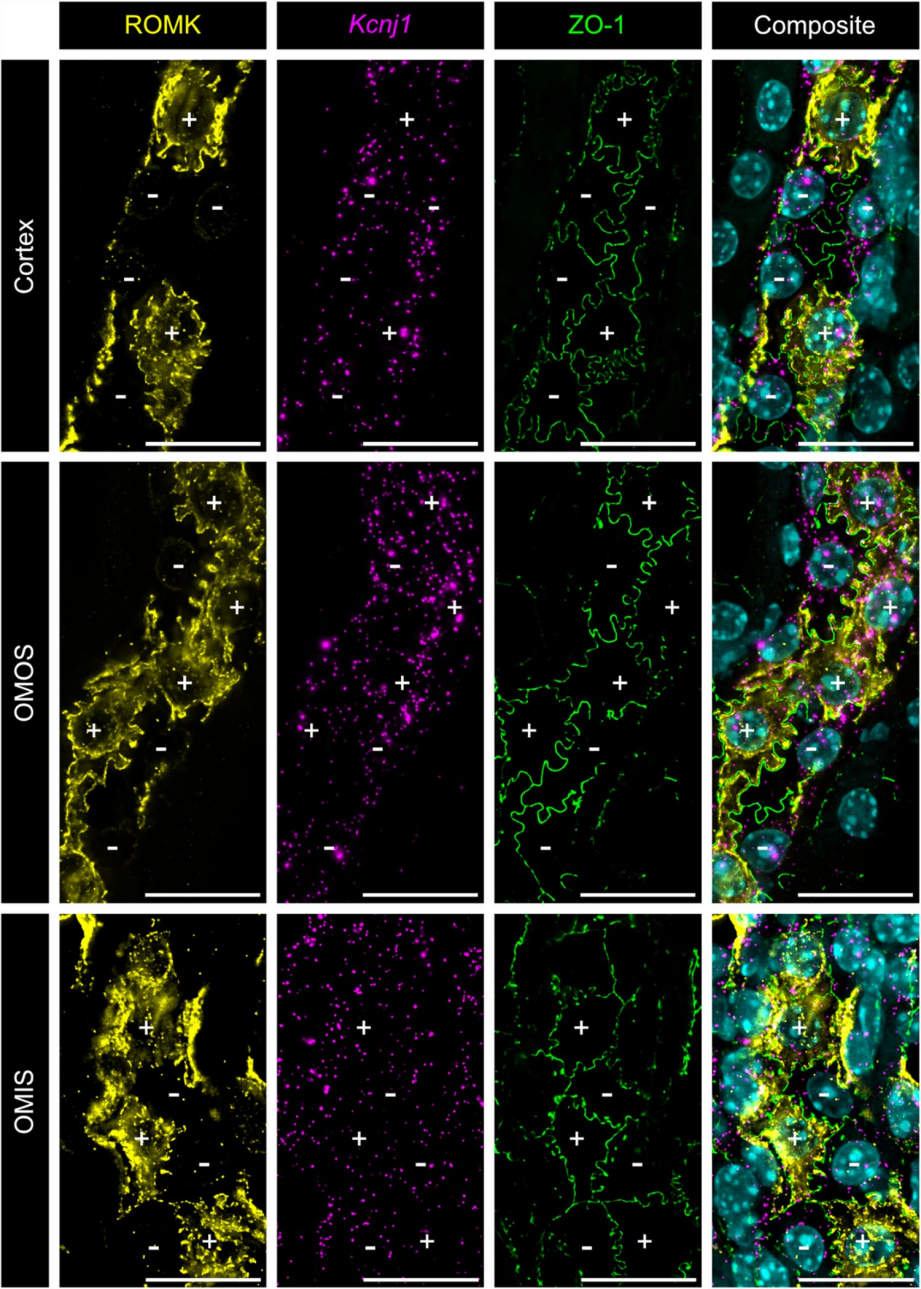
Co-detection of ROMK protein and *Kcnj1* mRNA. Immunofluorescent detection of ROMK (yellow) combined with RNAscope detection of *Kcnj1* mRNA (magenta) in TALs of the renal cortex, OMOS, and OMIS. Imaging sections represent the maximum projection from a z-stack starting at the cellular tight junctions, visualized by co-staining for ZO- 1 (green), up to the localization of the respective cell nuclei. Nuclei are stained with DAPI (cyan) in the composite images. + indicates cell with apical ROMK; − indicates cell without apical ROMK; scale bar = 20 μm

Dual immunostaining for NKCC2, a well-established TAL marker (Fig. 2A, C), and ROMK (Fig. 2B, D) confirmed that apical ROMK localization varies among TAL cells. While some cells exhibit strong apical ROMK expression, others lack it entirely. Overexposing the ROMK signal revealed weak perinuclear staining in both ROMK-positive and ROMK-negative TAL cells. Perinuclear ROMK staining, observed with two independent antibodies (Fig. 2D), suggests that ROMK is expressed in all TAL cell types but is trafficked to the apical membrane only in a subset of cells. Consistently, RNAscope detection of *Kcnj1* mRNA, which encodes ROMK, demonstrated that all TAL cells express *Kcnj1*, regardless of whether ROMK protein is present at the apical membrane. To better delineate individual TAL cells, we also performed experiments in which we co-stained for the TJ protein zonula occludens-1 (ZO-1), highlighting cell boundaries (Fig. 3).

### Ptger3 and Foxq1 mRNA expression correlate with apical ROMK presence

**Fig. 4:**
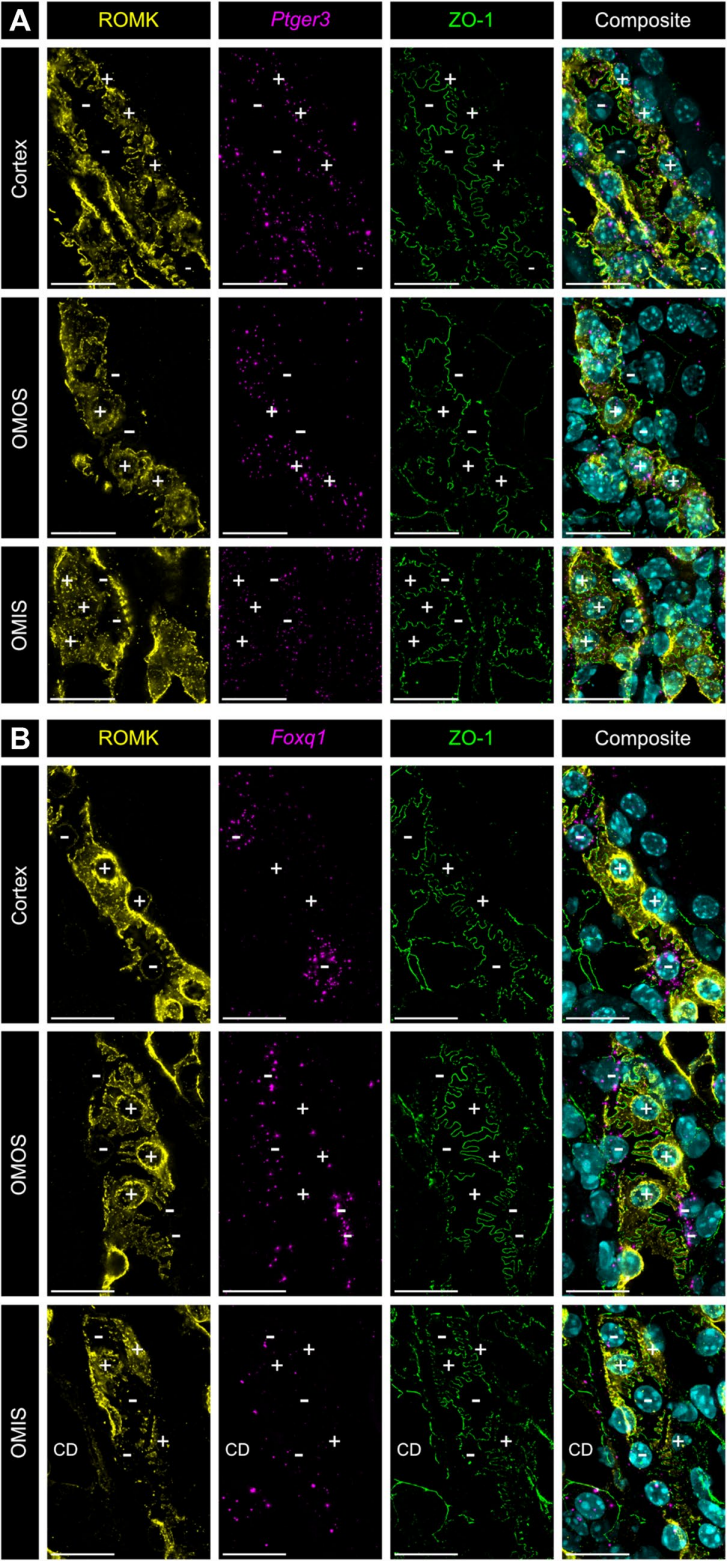
Co-detection of ROMK protein with *Ptger3* and *Foxq1* mRNA. Immunofluorescent detection of ROMK (yellow) combined with RNAscope detection of **A** *Ptger3* and **B** *Foxq1* (magenta) in TALs of the renal cortex, OMOS, and OMIS. Imaging sections represent the maximum projection from a z-stack starting at the cellular tight junctions, visualized by co-staining for ZO-1 (green), up to the localization of the respective cell nuclei. Nuclei are stained with DAPI (cyan) in the composite images. + indicates cell with apical ROMK; − indicates cell without apical ROMK; CD – collecting duct; scale bar = 20 μm

We hypothesized that the heterogeneity of apical ROMK abundance could reflect the presence of previously described molecularly distinct cortical TAL cell types [9]. Single-cell RNA sequencing (scRNA-seq) studies have shown that *Foxq1* and *Ptger3* are expressed in a mutually exclusive manner within cortical TAL cell populations C5 and C6, respectively [9]. To investigate this relationship, we used RNAscope in situ hybridization (ISH) to detect these marker mRNAs in combination with ROMK immunostaining. In the renal cortex and the outer medullary outer stripe (OMOS), all TAL cells with apical ROMK localization expressed the C6 marker *Ptger3*. In the outer medullary inner stripe (OMIS), apical ROMK localization remained heterogeneous, but all OMIS TAL cells expressed *Ptger3* mRNA (Fig. 4A). Conversely, the C5 marker *Foxq1* exhibited an opposing pattern. In the renal cortex and OMOS, TAL cells expressing *Foxq1* lacked apical ROMK, whereas all *Foxq1*-negative cells in these regions displayed apical ROMK localization. In the OMIS TAL, *Foxq1* expression diminished and became barely detectable in most TAL cells. However, in the subset of OMIS TAL cells where *Foxq1* remained detectable, ROMK was not targeted to the apical plasma membrane (Fig. 4B).

### Apical ROMK localization correlates with differential claudin and Kir4.1 expression

**Fig. 5:**
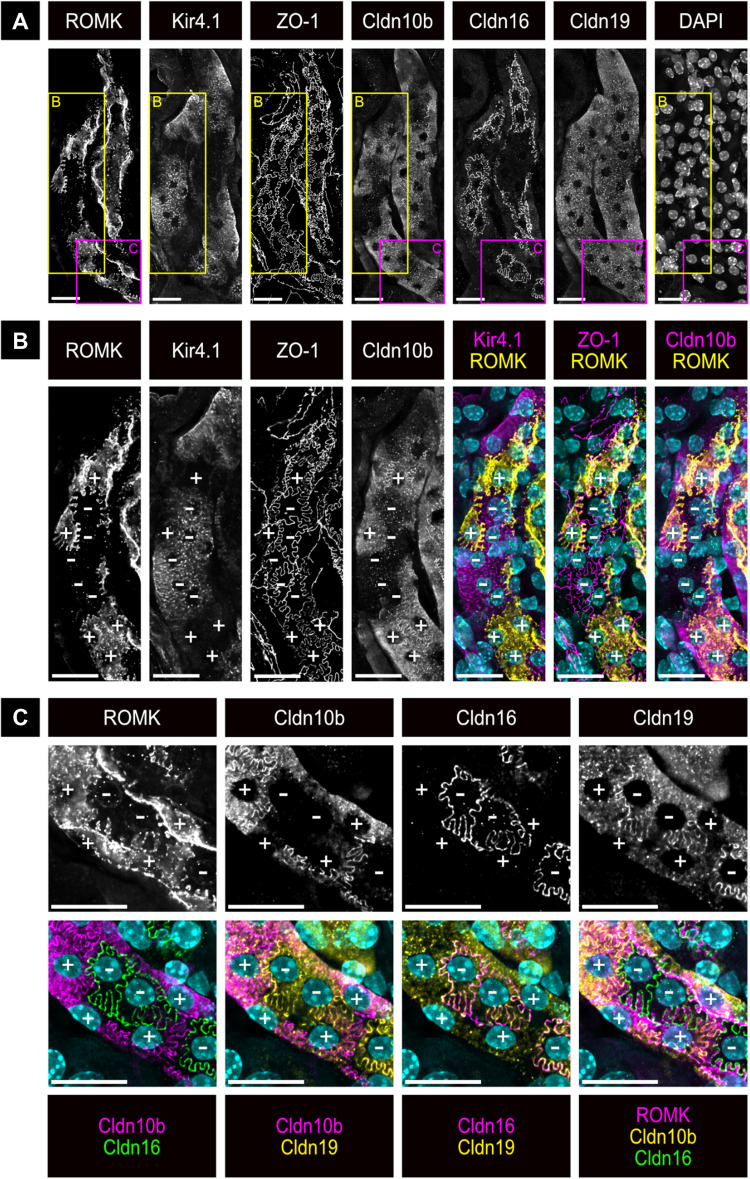
Co-detection of ROMK protein with Kir4.1, Cldn10b, Cldn16, and Cldn19 protein using 4i-multiplexing imaging. **A** Overview of a TAL in the renal cortex immunostained with antibodies against ROMK, Kir4.1, ZO-1, Cldn10b, Cldn16, and Cldn19. The imaging sections represent the maximum projection from a z-stack starting at the cellular tight junctions, visualized by co-staining for ZO-1, until the localization of the respective nuclei. Nuclei are stained with DAPI. **B**, **C** higher magnifications of the regions indicated in panel **A**. Composite images show co-labeling for two to three antigens, as indicated below the images; + indicates cell with apical ROMK; − indicates cell without apical ROMK; CD – collecting duct; scale bar = 20 μm

**Fig. 6:**
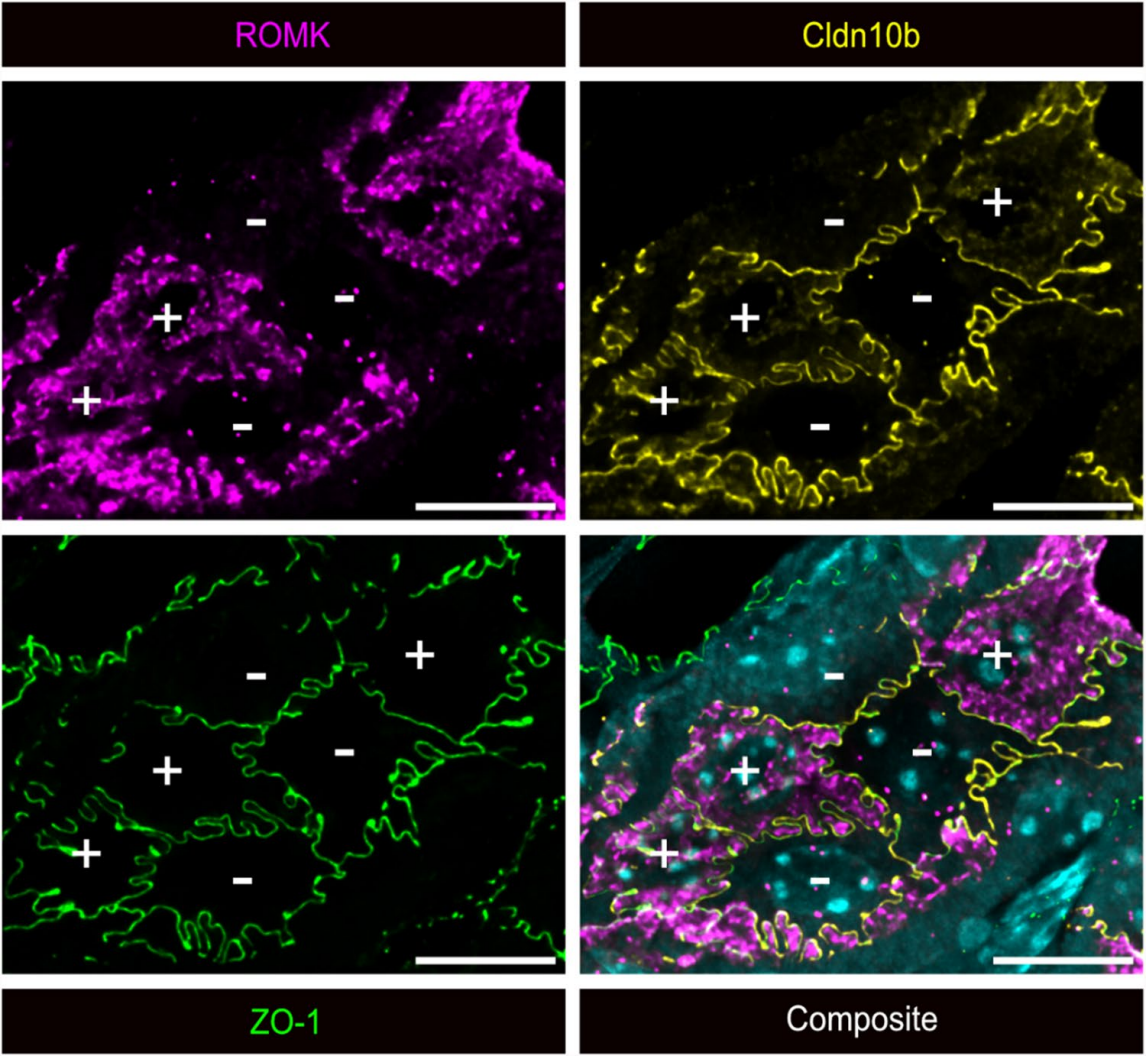
Co-detection of ROMK, Cldn10b, and ZO-1 in OMIS TAL. Immunohistochemical stainings of the TAL of the OMIS with antibodies against ROMK (magenta), Cldn10b (yellow) and ZO-1 (green) in consecutive cycles (Table 2). The imaging sections represent the maximum projection from a z-stack starting at the cellular tight junctions, visualized by co-staining for ZO-1 (green), until the localization of the respective cell nuclei stained with DAPI (cyan). + indicates cell with apical ROMK; − indicates cell without apical ROMK; scale bar = 10 μm

To further explore the relationship between apical ROMK localization and the expression of marker proteins specific to C5 and C6 TAL cell populations, we used the 4i multiplex technique [21]. This approach enables the precise immunodetection of multiple antigens within the same tissue section, even when antibodies originate from the same species (Fig. 1). For our analysis, we performed immunostaining for ROMK, Kir4.1, ZO-1, Claudin-10b (Cldn10b), Claudin-16 (Cldn16), and Claudin-19 (Cldn19), accompanied by nuclear staining with DAPI (Fig. 5A). High-magnification images of cortical TAL segments demonstrated that all Cldn10b-positive TAL cells exhibited apical ROMK localization, whereas all Kir4.1-positive TAL cells lacked apical ROMK (Fig. 5B). Additionally, immunostaining revealed that formed TJs are either Cldn10b-positive or Cldn16- and Cldn19-positive. The Cldn10b-positive TJ is only found between two cells with apical ROMK localization, while Cldn16/19-positive TJs are only formed around cells without apical ROMK. These findings indicate that the distribution of claudins along the cortical TAL is closely linked to the apical localization of ROMK. Similar observations were made for the OMOS TAL (data not shown). In contrast, in the OMIS TAL, all ZO-1-positive TJs also show immunostaining for Cldn10b. This indicates that all TJs, as marked by ZO-1, in the OMIS TAL are positive for Cldn10b, regardless of whether ROMK is localized apically or not (Fig. 6).

### ROMK is apically located in the macula densa (MD)

**Fig. 7:**
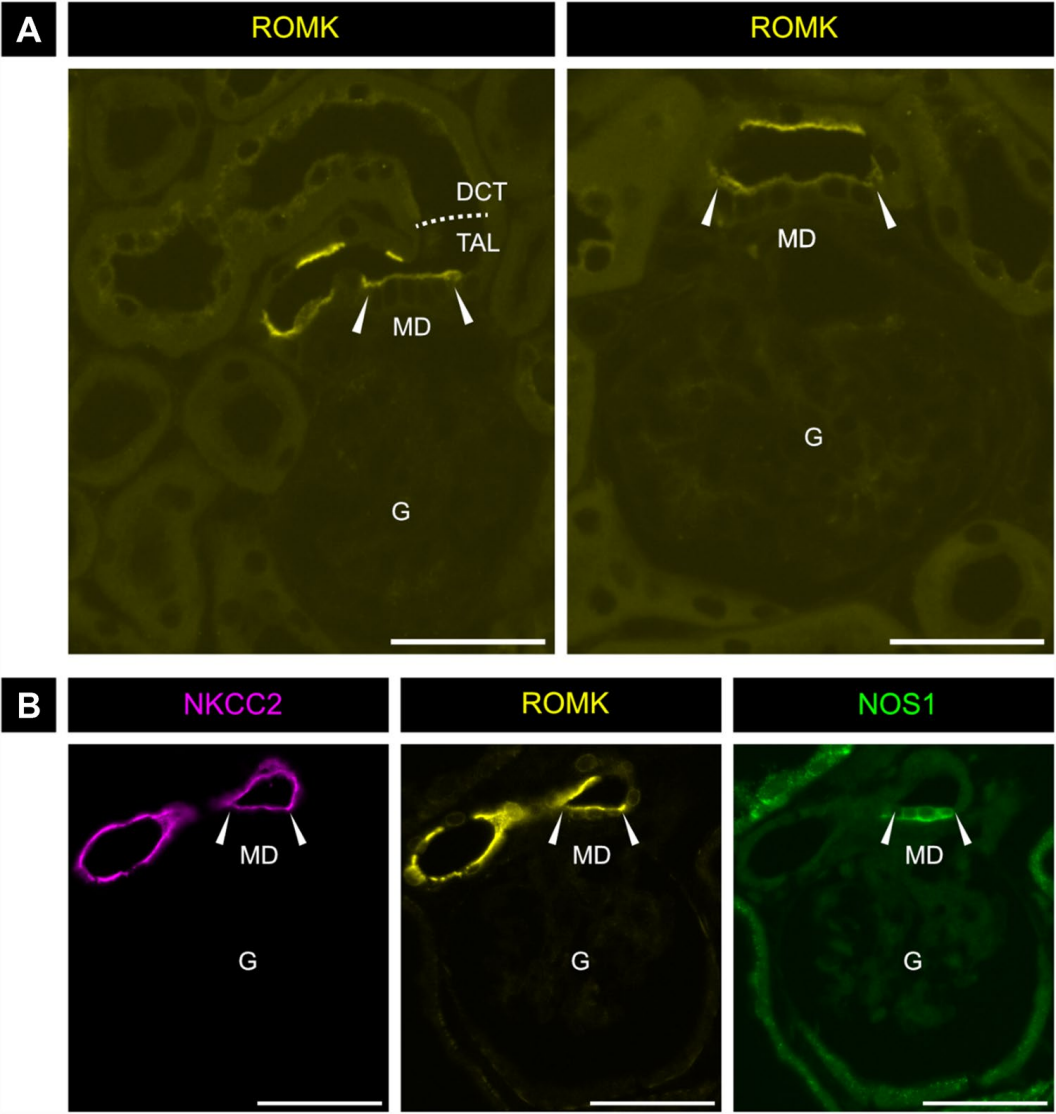
Immunodetection of ROMK protein at the macula densa. **A** Immunohistochemical stainings with the commercially available ROMK antibody (Table 2); **B** Immunohistochemical co-staining for NKCC2 (magenta), ROMK (yellow), NOS1 (green) using our previously published ROMK antibody (Table 2); arrowheads mark the region of the macula densa (MD); G – glomerulus; scale bar = 50 μm

The macula densa (MD) is a small cluster of densely packed, specialized TAL cells located at the vascular pole of the renal glomerulus. This distinctive position and cellular arrangement allow MD identification based on morphological criteria (Fig. 7A), which can be further confirmed by immunodetection of NOS1, a specific MD marker (Fig. 7B). Using these criteria, we specifically examined ROMK abundance and localization in the MD. Our analysis consistently showed that all MD cells exhibited apical ROMK. However, the intensity of apical ROMK immunostaining appeared weaker in MD cells compared to surrounding cortical TAL cells (Fig. 7). These findings were consistently observed using two independent antibodies.

### Quantification of apical TAL and MD surface positive for ROMK

**Fig. 8:**
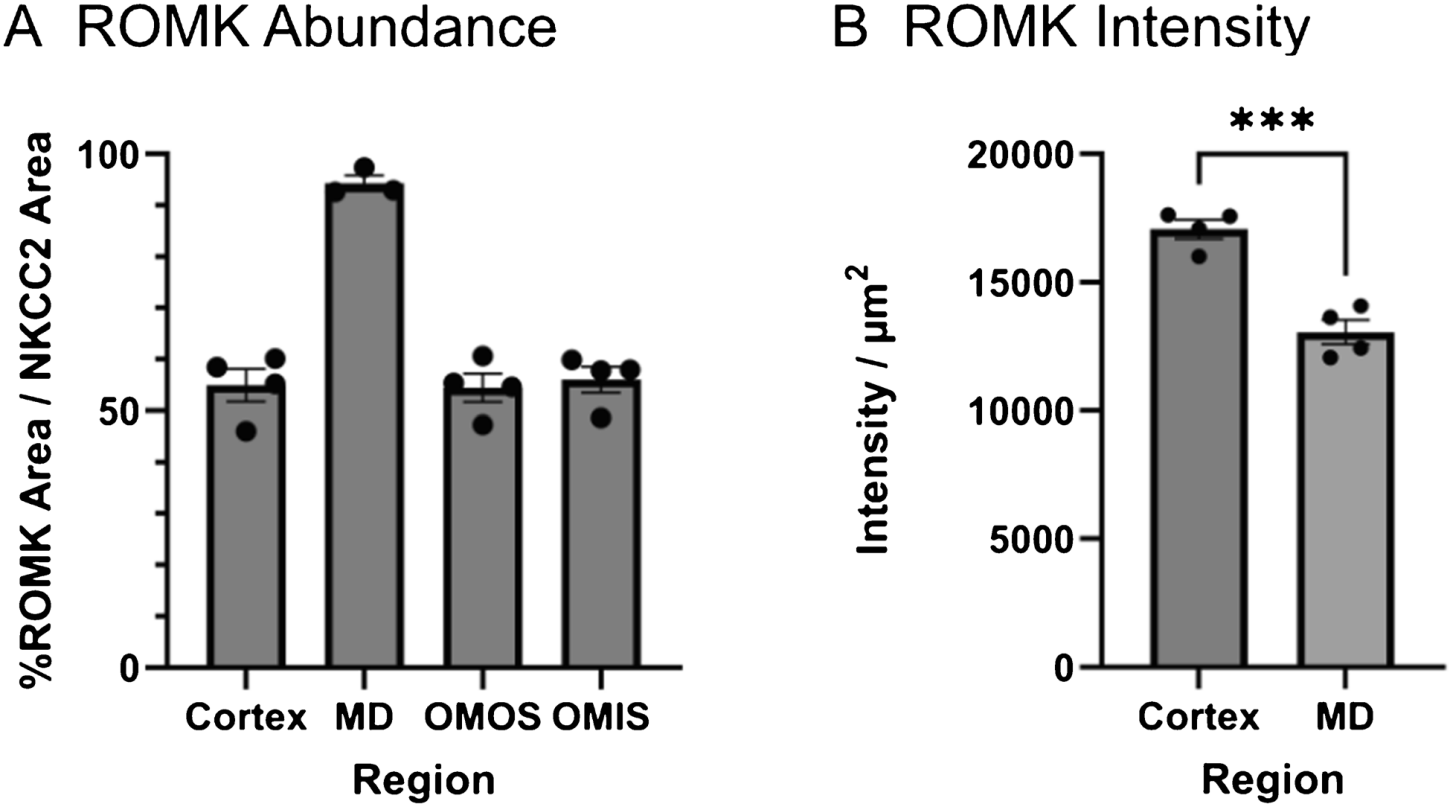
Quantification of ROMK immunostainings. **A** Percentage of the ROMK-positive apical area in NKCC2-positive TALs in the renal cortex, OMOS, OMIS, and in the macula densa (MD). **B** Intensity of ROMK immunofluorescence in the cortical TAL and the macula densa (MD) in arbitrary units. Data are presented as means ± standard error of mean; each data point per bar represents a different mouse

To quantify the proportion of the apical TAL cell surface expressing ROMK along different TAL segments – the OMIS TAL, the OMOS TAL, the cortical TAL, and the MD – we developed an automated, image-based quantification pipeline. As described in the “Methods” section, NKCC2 immunostaining was used as a general marker for the total apical membrane area of the TAL and MD, while NOS1 immunostaining identified the MD. Supervised machine learning (Ilastik & Fiji) was applied to convert immunofluorescence (IF) images into binary images, enabling pixel-by-pixel quantification of the ROMK-positive apical TAL membrane relative to the total NKCC2-positive apical surface (Fig. 1). Our analysis revealed that apical ROMK was present along 54.97% ± 3.15% of the cortical TAL, 54.49% ± 2.74% of the OMOS TAL, and 56.1% ± 2.5% of the OMIS TAL (*n* = 4 mice). In the MD, 94.3% ± 1.54% (*n* = 3) of the apical cell surface was ROMK-positive (Fig. 8A). Additionally, binary images were used to compare the signal intensity of apical ROMK staining in MD cells versus cortical TAL cells within the same histological section. Quantification confirmed that apical ROMK abundance in MD cells was significantly lower than in other cortical TAL cells exhibiting apical ROMK staining (Fig. 8B).

### Similar degree of apical ROMK heterogeneity in gene-modified mouse models with altered ion homeostasis and in mice of both sexes

To test if the apical ROMK abundance may vary between mice with altered ion homeostasis or with sex, we analyzed archived tissue from previous experiments performed in our laboratory. This included kidneys from age-matched wildtype mice and mice with a constitutive deletion of the NaCl cotransporter (NCC^−/−^) in the distal convoluted tubule (DCT), which have a positive calcium but negative magnesium and sodium balance [23, 34], and control mice (CnB1^fl/fl^) and mice with a DCT-specific deletion of the calcineurin regulatory subunit B1 (NCC^cre^ x CnB1^fl/fl^), which have acidosis and a negative calcium and magnesium balance [3]. Moreover, kidneys from adult male and female mice were analyzed. As indicated in Table 1, the groups of mice had different genetic backgrounds. Automated quantification of apical ROMK abundance in these mice revealed that the ratio of ROMK-positive vs. ROMK-negative apical cell surface corresponds well to the data shown in Fig. 8A and is quite stable across genotypes, sex, and genetic backgrounds (Table 1).

## Discussion

Heterogeneous apical ROMK expression along the TAL has been reported in multiple studies [24, 43, 46]. However, it remained unclear whether these differences in ROMK expression were due to the presence of distinct cell populations or merely reflected biological variability within a single cell type. A previous single-cell RNA sequencing study identified two distinct cortical TAL cell populations (C6 and C5), aside from the macula densa, characterized by the expression of either *Cldn10b* and *Ptger3* or *Cldn16* and *Foxq1*, respectively [9].

Strikingly, we found that apical ROMK abundance in the TAL of the renal cortex and the OMOS was exclusively present in cells expressing *Ptger3* (corresponding to the C6 cell population). Conversely, cells lacking apical ROMK in these segments were positive for *Foxq1* (corresponding to the C5 cell population). Additionally, apical ROMK localization in the cortical TAL was associated with TJs containing Cldn16 and Cldn19, while cells without apical ROMK formed TJs composed of Cldn10b. Furthermore, the basolateral K^+^ channel Kir4.1 (*Kcnj10*) was exclusively expressed in cells lacking apical ROMK. These findings strongly suggest that heterogeneous apical ROMK expression reflects the presence of distinct cell types rather than random variability.

Corresponding to the classification of the TAL cell subtypes in a previous snRNA-seq study on human kidney [42], we designated cells with apical ROMK as TAL cell type I (TAL-I) and cells without apical ROMK as TAL cell type II (TAL-II) corresponding to the C6 population and C5, respectively.

While this manuscript was in preparation, another study published as a preprint reported similar findings – specifically, the same heterogeneity in apical ROMK abundance along the TAL and its close association with TAL cell subtypes characterized by scRNA-seq [11]. In that study, the authors designated the C6 population with apical ROMK as α-cells (our TAL-I cells), and the C5 population lacks apical ROMK as β-cells (our TAL-II cells). Moreover, the authors designated the *Ptger3* negative but apical ROMK-positive TAL cells in the OMIS, γ-cells.

In the TAL, apical potassium secretion via ROMK generates the electrical gradient that drives paracellular sodium transport through Cldn10b and calcium/magnesium transport via Cldn16 and Cldn19 [15]. TJ composition varies along the TAL, with cells forming either Cldn16/19- or Cldn10b-containing junctions, but never both [6, 27]. Since apical ROMK is restricted to TAL-I cells, these cells likely generate the lumen-positive electrical gradient that facilitates not only paracellular sodium transport through Cldn10b-positive TJs formed by TAL-I cells, but also calcium/magnesium reabsorption through Cldn16/19-positive TJs formed by neighboring TAL-II cells.

**Fig. 9:**
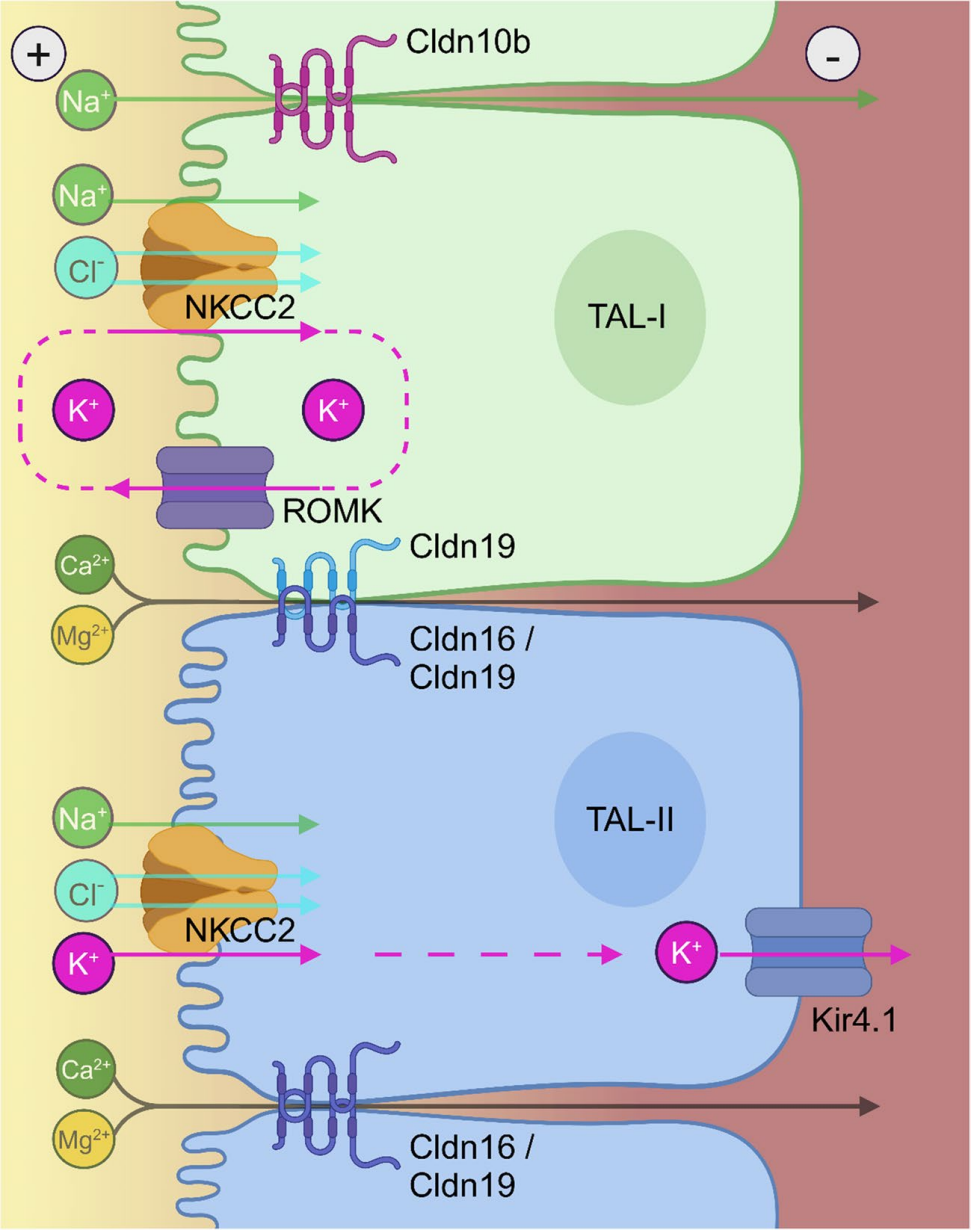
Schematic presentation of cell model. TAL-I cells with apical ROMK and TAL-II cells without apical ROMK are depicted in green and blue, respectively

Moreover, Kir4.1, a subunit of the basolateral 40-pS potassium channel, was shown to be expressed heterogeneously along the TAL [44]. Now, we show that Kir4.1 is restricted to TAL-II cells. Combined with the absence of apical ROMK in TAL-II cells, this suggests that TAL-II cells are well suited for net potassium reabsorption, whereas TAL-I cells likely exhibit little to no net potassium reabsorption (Fig. 9). These findings align well with electrophysiological studies in hamster TALs, which identified two distinct cell types with different electrophysiological properties: one with high apical and low basolateral potassium conductance and another with low apical and high basolateral potassium conductance [37].

A key observation of our study is that the ratio of TAL-I to TAL-II cells remains relatively constant across mouse strains, sex, and also along the TAL. However, while heterogeneous apical ROMK localization is also observed in the OMIS TAL, the *Ptger3* and *Foxq1* expression heterogeneity disappears in the OMIS (Fig. 4). Similarly, a study by Bleich and colleagues demonstrated that all TAL cells in the OMIS exclusively possess Cldn10b-positive TJs and lack Cldn16/19 expression [6], consistent with the function of the OMIS TAL in sodium reabsorption but not calcium or magnesium reabsorption [6, 12, 13, 33, 36]. We confirmed that all OMIS TAL cells have Cldn10b-positive TJs and that they are formed not only by cells with apical ROMK, but also by TAL cells without apical ROMK (Fig. 6). Based on functional studies of TAL subsegments microdissected from either the kidney cortex or the OMIS, the literature commonly used the terms cortical TAL (cTAL) and medullary TAL (mTAL) to differentiate these segments [4, 13, 28]. Our data further support the notion that the OMOS TAL is molecularly and functionally similar to the cTAL, yet distinct from the OMIS TAL [4]. This is further supported by enriched scRNA sequencing data from Demirci and co-workers, who showed that indeed the OMIS TAL cells without apical ROMK are molecularly distinct from the cortical and OMOS TAL cells without apical ROMK [11]. Interestingly, TAL cells with apical ROMK appear to have a similar molecular make-up in OMIS, OMOS, and cortical TAL [11].

Despite regional differences in apical ROMK expression along the TAL, our quantification analysis revealed that nearly all macula densa (MD) cells exhibit apical ROMK expression (94.3% ROMK-to-NKCC2 ratio). This suggests that ROMK-mediated potassium recycling plays a crucial role in NKCC2 function at the MD, influencing sodium and chloride sensing, as well as tubuloglomerular feedback.

Notably, TAL-II cells lack apical ROMK but express *Kcnj1* mRNA and display perinuclear ROMK localization, raising the question of whether these cells can translocate ROMK to the apical membrane under conditions requiring enhanced paracellular ion reabsorption. TAL ion transport is regulated by multiple factors, including hormones (e.g., vasopressin, parathyroid hormone, glucagon), dietary sodium, potassium and water intake, and extracellular calcium [14, 25, 28, 40]. Our analysis of archived tissue from gene-modified mouse models with altered calcium, magnesium, sodium, and acid–base balance did not reveal significant differences in the ratio of ROMK-positive to ROMK-negative TAL surface area between the control and knockout mice. However, additional experiments are needed to further address this issue. Future studies should also investigate whether TAL-I and TAL-II cells can undergo transdifferentiation and identify regulatory factors involved. Other limitations of our study include that we did not analyze whether the ratio of TAL-I to TAL-II cells may change with age or between species. Moreover, our study relies only on histological imaging and does not include functional studies that further analyzed the cellular heterogeneity and tested for the proposed cell models.

In conclusion, our findings indicate that the mouse cortical and OMOS TAL consist of three distinct cell subtypes: TAL-I cells, TAL-II cells, and MD cells. The OMIS TAL is composed of cells, which homogenously express *Cldn10b* and *Ptger3* but still show the same heterogeneity of apical ROMK as the cortical and OMOS TAL. This cellular heterogeneity likely supports specialized roles in ion transport along the TAL, contributing to its physiological function. Future studies should explore whether human TAL cells exhibit a similar heterogeneity and investigate the mechanisms regulating TAL-I and TAL-II cell differentiation.

## Data Availability

Described data will be made available on reasonable request.
